# Optical, electrical and photoresponse data of flexible and high-performing NiO/ZnO ultraviolet photodetector

**DOI:** 10.1016/j.dib.2018.01.064

**Published:** 2018-02-20

**Authors:** Malkeshkumar Patel, Sung-Ho Park, Joondong Kim

**Affiliations:** a Department of Electrical Engineering, Incheon National University, 119 Academy Rd. Yeonsu, Incheon 406772, Republic of Korea; b Photoelectric and Energy Device Application Lab (PEDAL), Multidisciplinary Core Institute for Future Energies (MCIFE), Incheon National University, 119 Academy Rd. Yeonsu, Incheon 406772, Republic of Korea

## Abstract

In this work, the fabrication process flow of ZnO/NiO heterojunction device on a PET substrate, optical properties, physical properties and photoresponses presented (Patel and Kim, 2017) [1]. Absorption coefficient and Tauc plots of ZnO and NiO samples are summarized. Digital photograph of flexible NiO/ZnO/ITO device on a PET substrate is presented. Surface morphologies of ITO on PET, polycrystalline ZnO on ITO/PET, and nanocrystalline NiO on ZnO/ITO/PET is presented with a demonstration of scissor-cut design. NiO/ZnO/ITO/PET photoelectric device has advantages of large-scale production and light-weight. Transmittance, reflectance and absorbance dataset of the native PET substrate (100 μm thick) is summarized. Photoresponses of the transparent (NiO/ZnO/ITO/PET) device with bias modulations including the rising edge and falling edge are included in this article.

**Specifications Table**

**Table:** 

Subject area	*Physics, Electrical Engineering*
More specific subject area	*Zero-biased ultraviolet photodetector*
Type of data	*Figures*
How data was acquired	*UV-visible spectrophotometer (UV-2600, Shimadzu)*
	*Field emission scanning electron microscope (FESEM, JOEL, JSM_7800 F)*
	*Digital photograph*
	*Potentiostat/Galvanostat (ZIVE SP1, WonA Tech, Korea)*
Data format	*Analyzed*
Experimental factors	*UV-visible, sample mounted on the diffused integrated sphere; scan range 1200–250 nm;*
	*FESEM (samples grown on the PET substrate)*
	*Digital photograph (Ambient light condition)*
	*Transient photoresponse: UV light source 365 nm, sample interval 40 μs*
Experimental features	*Transparent, flexible NiO/ZnO/ITO/PET high speed zero-biased ultraviolet photodetector*
Data source location	*Incheon National University, Incheon-406772, Korea*
Data accessibility	*The data are with this article*

**Value of the data**

• Photograph of the prepared NiO/ZnO/ITO/PET devices for the transparent feature and scissor cut design.
• *Absorption coefficient and Tauc plots of ZnO and NiO samples.*
• Surface morphology of large area grown ZnO, NiO and ITO films on the PET substrate.
• Transient ultraviolet photoresponses and its bias dependents of NiO/ZnO/ITO/PET device.

## 1 Data

**Fig. 1:**
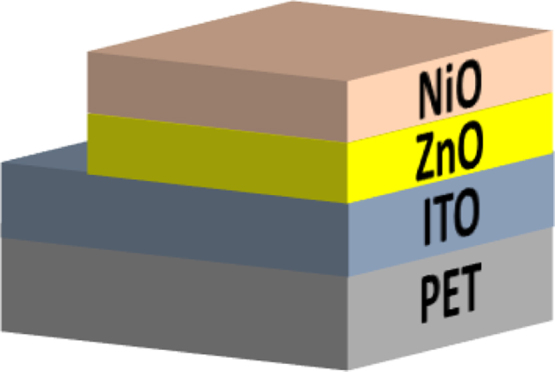
Process flows of fabrication for the transparent photoelectric device on a PET substrate. ITO, ZnO, and NiO layers were sequentially deposited under in-line sputtering processes without breaking a vacuum condition. Due to the use of the flexible PET substrate, all metal oxides were deposited at room temperature.

**Fig. 2:**
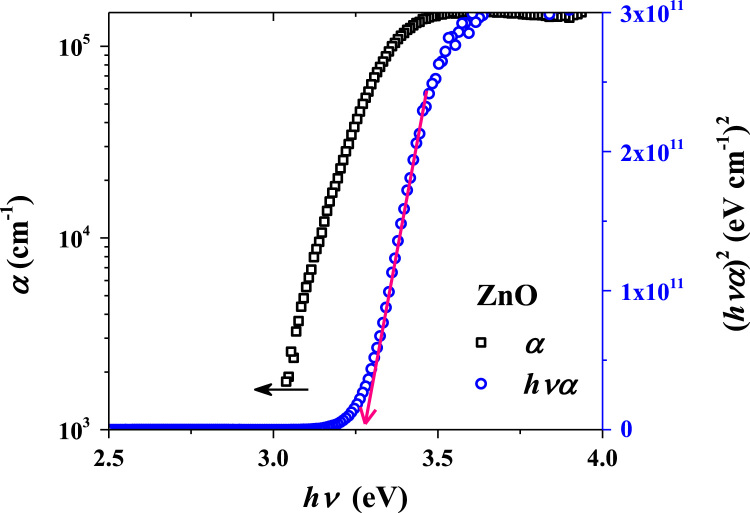
Absorption coefficient and Tauc plot of ZnO film on a PET substrate. High slope of the α values near bandgap to indicate the exciton induced photon absorption in the ZnO. Tauc plot indicates a direct optical bandgap value of 3.28 eV.

**Fig. 3:**
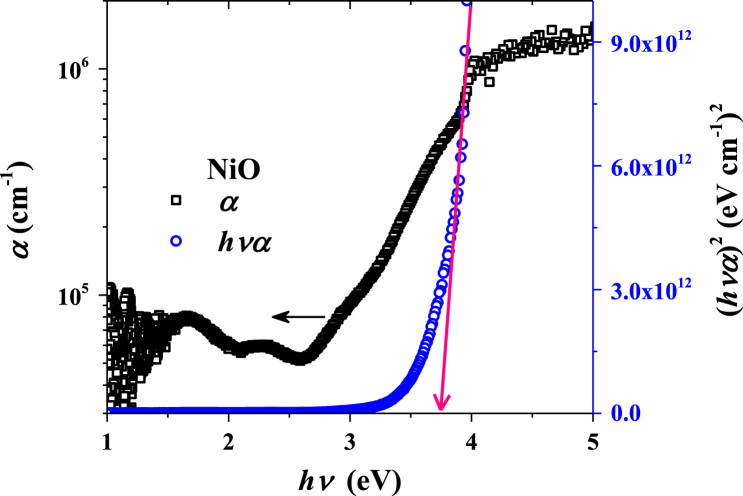
Absorption coefficient and Tauc plot of NiO film on a PET substrate. High absorption coefficient values confirm the quantum dot nature of NiO. Tauc plot indicates a direct optical bandgap value of 3.8 eV.

**Fig. 4:**
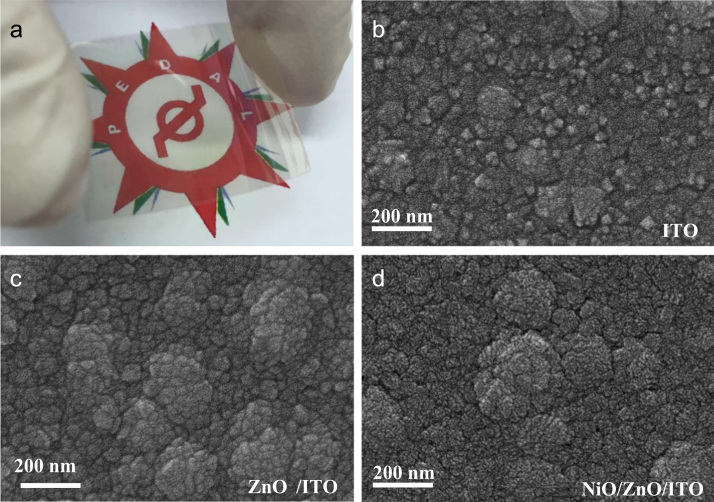
(a) Digital photograph of flexible NiO/ZnO/ITO device on a PET substrate. Surface morphologies of (b) ITO on PET, (c) Polycrystalline ZnO on ITO/PET, and (d) Nanocrystalline NiO on ZnO/ITO/PET.

**Fig. 5:**
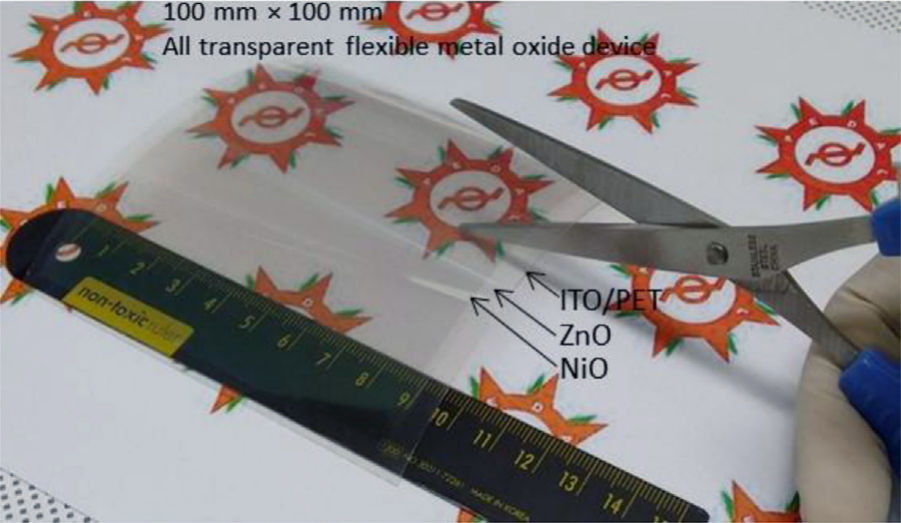
A demonstration of scissor-cut design. NiO/ZnO/ITO/PET photoelectric device has advantages of large-scale production and light-weight.

**Fig. 6:**
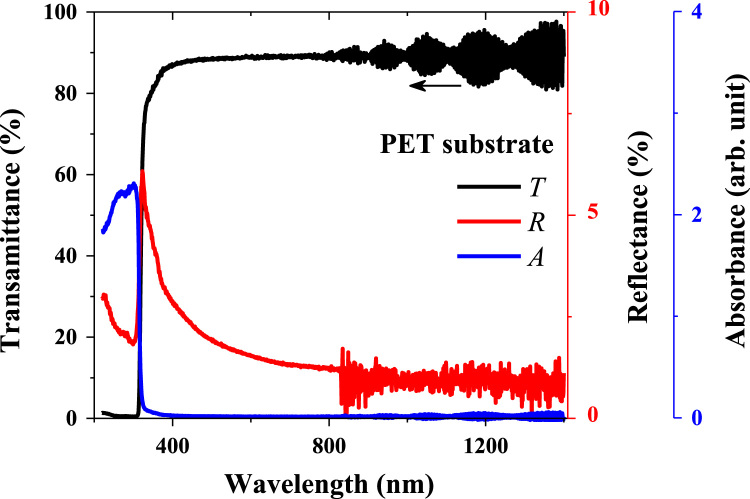
Transmittance, reflectance and absorbance properties of the native PET substrate (100 μm thick).

**Fig. 7:**
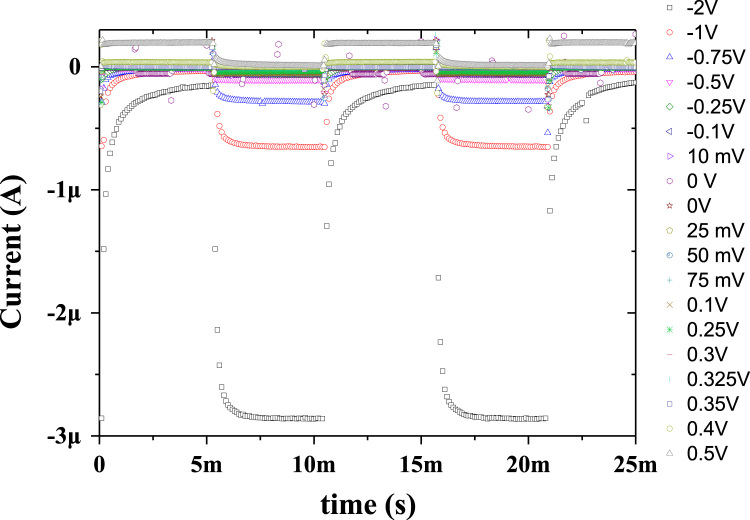
Photoresponses of the transparent (NiO/ZnO/ITO/PET) device with bias modulations.

**Fig. 8:**
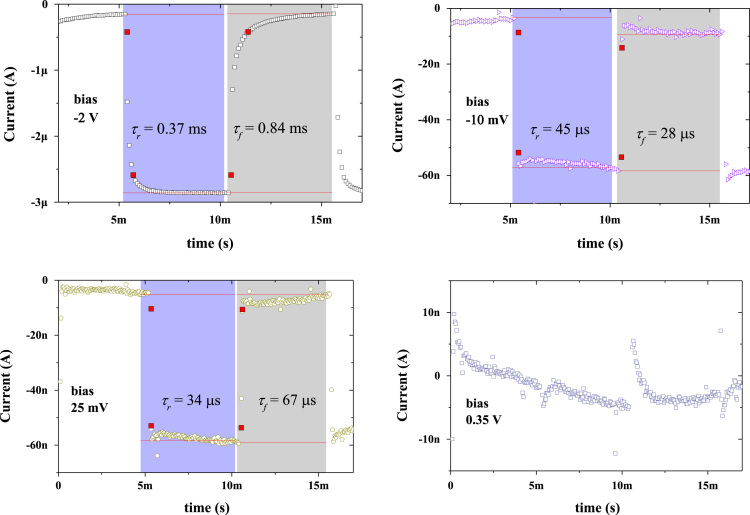
Transient photoresponse analysis of the transparent (NiO/ZnO/ITO/PET) device.

Fig. 1 shows the schematic of the NiO/ZnO/ITO/PET device including the fabrication process flow. Absorption coefficient and Tauc plot of ZnO and NiO film fabricated on the PET substrate is shown in Fig. 2, Fig. 3, respectively. Digital photograph of the flexible NiO/ZnO/ITO device on a PET substrate, surface morphologies of ITO, polycrystalline ZnO on ITO/PET, and nanocrystalline NiO on ZnO/ITO/PET are presented in Fig. 4. A demonstration of scissor-cut design. NiO/ZnO/ITO/PET photoelectric device has advantages of large-scale production and light-weight is presented in Fig. 5. Optical properties of native PET substrate is measured and its transmittance, absorbance and reflectance spectra as a function of photon wavelength is shown in Fig. 6. Photoresponses of the transparent (NiO/ZnO/ITO/PET) device with bias modulations including their analysis is presented in the Fig. 7, Fig. 8.

## 2 Experimental design, materials and methods

### 2.1 Sample preparation

Commercial PET was used as the flexible substrate and was cleaned according to Ref. [1]. ITO film was formed using DC sputtering (Power 300 W, pressure 5 mTorr, Ag flow 50 sccm, substrate rotation 5 rpm) at room temperature. The ZnO film was formed using the RF magnetron sputtering. Conditions for preparing ZnO sample is as follows.

**Table:** 

*Target*	ZnO (Ø4 inch, purity 99.999%)
*RF power*	300 W
*Gas/flow rate*	Ar/50 sccm
*Deposition pressure*	5 mTorr
*Temperature*	Room temperature
*Substrate rotation*	5 rpm

NiO film was formed using the DC reactive sputtering. Conditions for preparing NiO sample are as follows.

**Table:** 

*Target*	Ni (Ø4 inch, purity 99.999%)
*DC power*	50 W
*Gas/flow rate*	Ar/50 sccm, O_2_/1 sccm
*Deposition pressure*	3 mTorr
*Temperature*	Room temperature
*Substrate rotation*	5 rpm

### 2.2 Sample characterizations

Optical properties of ZnO and NiO films fabricated on the PET substrate were measured using the UV-visible spectrophotometer. The measured transmittance and reflectance spectra were used to estimate the absorption coefficient as well described in [1]. The thickness profiles of ZnO and NiO thin films were estimated using the energy dispersive spectroscopy (elemental line profile) in our previous work [1]. The calculated absorption coefficient as a function of photon energy (hv) as shown in Fig. 2, Fig. 3 are corresponding to the ZnO and NiO samples, respectively. On the secondary axis, Tauc plot is presented, which shows the band gap value. Surface morphology of fabricated films of NiO on ZnO/ITO/PET, ZnO on ITO/PET and ITO on PET were measured using FESEM and these images are shown in Fig. 4 including the photograph of NiO/ZnO/ITO/PET flexible device. A demonstration of scissor-cut design, NiO/ZnO/ITO/PET (device area 100 mm × 100 mm) photoelectric device has advantages of large-scale production and light-weight is shown in Fig. 5. Photoresponses of the transparent (NiO/ZnO/ITO/PET) device with bias modulations is shown in Fig. 6. These dataset were measured using Chronoamperometry and the pulsed UV (365 nm) light. The bias was applied from − 2 V to 0.5 V and each case were analyzed for estimating the rise time (*τ*_r_) and fall time (*τ*_f_) of the photoresponse as shown in Fig. 7. Built in function of Origin tool was applied to estimate the *τ*_r_ and *τ*_f_ values.
