# Supplementary material for: Optical, electrical and photoresponse data of flexible and high-performing NiO/ZnO ultraviolet photodetector
Source: Data Brief. 2018 Feb 20;17:520–5. doi: 10.1016/j.dib.2018.01.064 (PMC5988381; doi:10.1016/j.dib.2018.01.064)
Supplement: Supplementary file 1 — Transparency document [file mmc1.docx]

***Conflicts of Interest Statement***

Re: DIB-D-17-01035

Title: Optical, electrical and photoresponse data of flexible and high-performing NiO/ZnO ultraviolet photodetector

We declare that this manuscript is original, has not been reported before, and is not currently being considered elsewhere. We also confirm that there is no known conflict of interest regarding this manuscript and its publication. The manuscript has been approved by all named authors.

Sincerely yours,


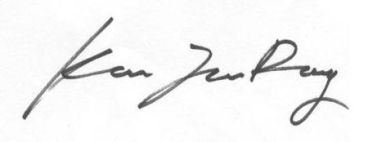


Joondong Kim

Joondong Kim, Ph.D./Professor

Department of Electrical Engineering,

Incheon National University

E-mail: joonkim@ incheon.ac.kr

Phone: +82-32-835-8770; fax: +82-32-835-0773
